# Increased Serum Levels of IL-17A and IL-23 Are Associated with Decreased Vitamin D3 and Increased Pain in Osteoarthritis

**DOI:** 10.1371/journal.pone.0164757

**Published:** 2016-11-07

**Authors:** Alireza Askari, Mohammad Mehdi Naghizadeh, Reza Homayounfar, Abbas Shahi, Mohammad Hosein Afsarian, Abbas Paknahad, Derek Kennedy, Mohammad Reza Ataollahi

**Affiliations:** 1 Noncommunicable Diseases Research Center, Fasa University of Medical Sciences, Fasa, Iran; 2 Student Research Committee, Fasa University of Medical Sciences, Fasa, Iran; 3 Student Research Committee, Hormozgan University of Medical Sciences, Bandar Abbas, Iran; 4 School of Natural Sciences, Eskitis Institute for Drug Discovery, Griffith University Nathan, Queensland, Australia; Queen Mary University of London, UNITED KINGDOM

## Abstract

**Introduction:**

Osteoarthritis (OA) is the most common type of arthritis and proinflammatory cytokines have been considered as the main etiologic factor in the pathogenesis of the disease. Serum levels of cytokines, that are associated with innate immunity and TH1 cells, have been analyzed in OA patients, however, there is limited research that profiles cytokines associated with Th17 cells and their relation to vitamin D3 and pain.

**Material and methods:**

The sera from 131 patients with OA and 262 healthy controls were evaluated for serum levels of IL-17A, IL-21, IL-23 and vitamin D3 using ELISA.

**Results:**

Serum levels of IL-17A, and IL-23 were statistically higher in OA patients than in healthy controls, while IL-21 and vitamin D3 were significantly lower in OA patients when compared to controls. A significant positive correlation was found between the serum levels of IL-17A and IL-23 using WOMAC pain scores and vitamin D3 serum levels.

**Discussion:**

The results suggest that IL-17A plays a significant role in OA pathogenesis and the induction of pain. Decreased serum levels of vitamin D3 may reflect a positive role played by the factor in the regulation of immune responses in OA patients.

## Introduction

Osteoarthritis (OA), which is the most common type of arthritis, is a chronic and degenerative disease of synovial joints. In addition to articular cartilage breakdown, other joint tissues such as synovial membranes and subchondral bone are also involved in the progression of the disease [1, 2]. Pain control and improvement of joint function is the main goal in the treatment of osteoarthritis. The usual drug treatments for this disease are nonsteroidal anti-inflammatory drugs (NSAID), analgesics, topical corticosteroids and injection of hyaluronic acid into the affected joint [3]. The exact etiology of osteoarthritis is yet to be clarified, however, several studies have shown that inflammation at the early stages of the disease appears to play a role in the development and progression of the disease [3]. Not surprisingly, proinflammatory cytokines like IL-1β, TNF and IL-6, play key roles as the main mediators of joint tissue catabolism in OA [4–6].

Among the proinflammatory cytokines, IL-17A, IL-21 and IL-23 are considered as those which play crucial roles in the induction of local inflammation [7] and cartilage destruction diseases such as rheumatoid arthritis (RA) [8]. Moreover, the *in vitro* effect of IL-17A on chondrocytes from OA patients and subsequent cytokine release and cartilage destruction has also been reported [9]. There are similarities between RA and OA with regards to inflammation and joint destruction [8], hence, it may be hypothesized that proinflammatory cytokines may play a role in the pathogenesis of both diseases.

Vitamin D3 has significant effects on bones through its role in calcium and phosphor metabolism but it also has immunomodulatory properties which enable vitamin D3 to regulate expression of several immune related molecules [10]. Therefore, vitamin D3 has the potential to regulate immune responses that can attenuate proinflammatory diseases including autoimmune diseases. In accord with this, decreased serum levels of vitamin D3 in the proinflammatory based diseases, including autoimmune diseases, have been reported in several studies [11, 12]. However, information regarding the status of vitamin D3 in OA is a controversy [13]. For example, increased and decreased expression of vitamin D3 in OA has been reported by several investigators [13–15].

The aim of the present study was to determine the serum levels of IL-17A, IL-21, IL-23 and vitamin D3 in a population of Iranian OA patients and their correlation with clinical manifestations of OA. The second aim of this study was to examine the relationship between serum levels of vitamin D3 and the proinflammatory cytokines.

## Material and Method

### Subjects

This cross-sectional study was performed from the 1^st^ of January 2015 to the 31^st^ of December 2015 on 131 patients (24 males and 107 females) with knee OA who were referred to the Orthopedic Department of Hamza Clinic, Fasa, Iran. All patients fulfilled the American College of Rheumatology (ACR) criteria for OA diagnosis [16]. Patients with symptoms of other chronic inflammatory diseases, diabetes, a history of corticosteroid treatment within the previous month prior to recruitment to the study and patients with other forms of arthritis were excluded from the study. The control group contained 262 sex matched healthy subjects without any sign of arthritis and other orthopedic disease. This study was approved by the Human Ethics Committee of the Fasa University of Medical Sciences. All participants were informed about the study procedure and all patients signed an inform consent before blood sampling. Blood samples were taken from the participants and sera was separated immediately and then kept at -20°C for further analysis.

### Function and pain assessment

To assess pain in patients with knee OA, the Western Ontario McMaster University Osteoarthritis Index (WOMAC) questionnaire was used. This questionnaire involves functional activities and consists of questions regarding pain during horizontal motion, walking up and down stairs, sitting, standing, lying down, and pain at night (http://www.rheumatology.org/I-Am-A/Rheumatologist/Research/Clinician-Researchers/Western-Ontario-McMaster-Universities-Osteoarthritis-Index-WOMAC). A scale of 0–4 is used for scoring, where 0 means no pain and higher scores represent more severe pain. The total range of the WOMAC pain score is from 0 to 20.

### Cytokine assays

The sera were tested for the presence of IL-17A, IL-21 and IL-23 using a commercial ELISA kit (eBioscience, USA) according to the manufacturer's recommendations. The sensitivity of the ELISA kit for the detection of IL-17A, IL-21 and IL-23 was 4pg/ml, 8pg/ml and 15pg/ml, respectively.

### Vitamin D3 assay

Serum levels of vitamin D3 were evaluated using a commercial enzyme linked fluorescent assay (ELFA) kit from Vidas Company (Paris, France) according to the manufacturer's guidelines.

### Statistical analysis

All statistical analyses were performed using SPSS 20 software (Chicago, IL). Age, BMI, serum levels of IL-21, IL-23, IL-17A and vitamin D3 were compared between groups using independent sample t tests. The Chi square test was used for comparisons of gender between the two groups. Correlation was computed by use of a Pearson test. A logistic regression was used to determined predictor(s) of OA. In this analysis OA served as the dependent variable, and cytokines, Vitamin D3, BMI, age and gender were considered as the independent variables. Data was presented showing the median and mean ± standard deviation and P-values less than 0.05 were considered significant.

## Results

Statistical analysis revealed that there were statistically significant differences in age, BMI and WOMAC pain scores between the OA patients and healthy controls, while the two groups were matched regarding sex (Table 1).

**Table 1:** Status of sex, age, BMI and WOMAC in OA patients and healthy controls.

	OA (131)	Control (262)	P.value
Age	52 ± 8	55 ± 9	0.007
Sex (m/f)	24/107	48/214	0.551
BMI	28.24 ± 2.19	25.97 ± 2.38	< 0.001
WOMAC	15.11 ± 3.24	5.50 ± 2.80	< 0.001

Table illustrates that OA patients and healthy controls were significantly differ regarding age, BMI and WOMAC, while sex were match between groups.

**Fig 1:**
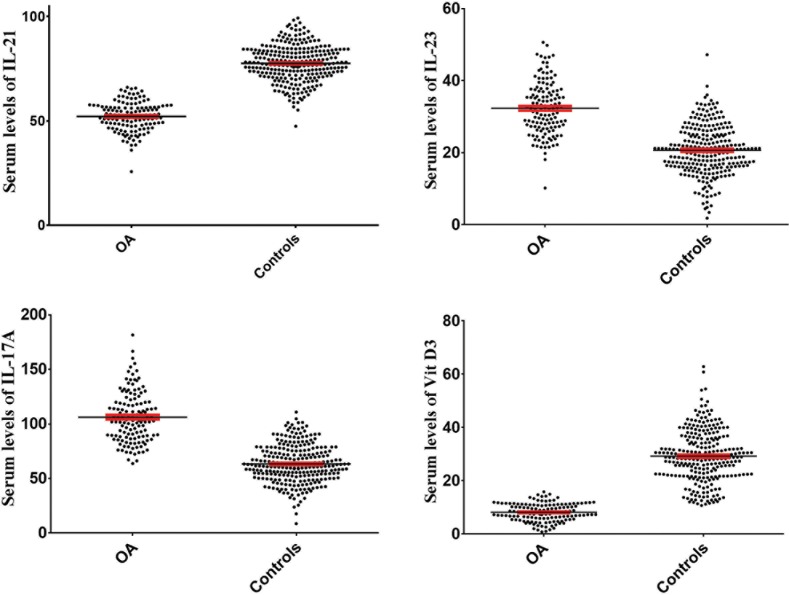
Serum levels of IL-21, IL-23, IL-17A and vitamin D3 in OA patients in comparison to healthy controls. Statistical analysis revealed that serum levels of IL-23 and IL-17A were significantly increased, while, IL-21 and vitamin D3 were decreased in OA patients in comparison to healthy controls.

The results also revealed that serum levels of IL-23 (P< 0.001) and IL-17A (P< 0.001) were significantly increased in OA patients (32.49 ± 7.12 and 106.24 ± 23.03 pg/mL, respectively) when compared with healthy controls (20.62 ± 6.86 and 63.46 ± 17.40 pg/mL, respectively). Serum levels of IL-21 in OA patients and healthy controls were 52.14 ± 7.04 and 77.58 ± 8.80 pg/mL, respectively, which indicates that IL-21 was significantly decreased in OA patients (P< 0.001). Vitamin D3 levels were also significantly (P< 0.001) decreased in OA patients (8.14 ± 3.33) in comparison to healthy controls (29.21 ± 9.75). Logistic regression modelling which excluded the interfering factors, age and BMI, also confirmed these results (Table 2). The Fig 1 illustrates the serum levels of IL-21, IL-23, IL-17A and vitamin D3 in OA patients and healthy controls.

**Table 2:** Analysis of variables using logistic regression model.

	IL-17 (Adj. R2 = 0.556)		IL-21 (Adj. R2 = 0.722)		IL-23 (Adj. R2 = 0.464)	
	B*	P-value	B*	P-value	B*	P-value
State (OA)	36.948	< 0.001	-24.198	< 0.001	9.780	< 0.001
Gender (female)	4.671	0.077	0.949	0.381	0.035	0.970
Age	0.101	0.424	-0.036	0.489	0.054	0.223
BMI	2.077	< 0.001	-1.301	< 0.001	0.956	< 0.001
Vitamin D	- 0.065	0.578	-0.077	0.113	-0.003	0.947

Table illustrates that the differences regarding serum levels of the cytokines and vitamin D3 are significant too after exclusion of age and BMI as interfered factors.

B*: Unstandardized Regression Coefficients beta.

Evaluation of correlation between WOMAC pain scores and serum levels of IL-21, IL-23, IL-17A and vitamin D3 using a Pearson correlation test revealed that WOMAC pain scores have a significant positive correlation with IL-23 and IL-17A and a significant negative correlation with IL-21 and vitamin D3 (Table 3). The data presented in Table 3 also demonstrated that vitamin D3 had a positive correlation with IL-23 and IL-17A.

**Table 3:** Correlation between WOMAC pain scores, IL-21, IL-23, IL-17A and vitamin D3 in OA patients.

		WOMAC pain scores	IL-21	IL-23	IL-17A	Vitamin D3
WOMAC pain scores	Pearson correlation	1	-0.175	0.306	0.315	-0.219
	P value		0.045	<0.001	<0.001	0.012
	Number	131	131	131	131	131
Vitamin D3	Pearson correlation	-0.219	0.064	-0.441	-0.406	1
	P value	0.012	0.469	<0.001	<0.001	
	Number	131	131	131	131	131

Table illustrates that WOMAC pain scores have significant positive correlations with IL-23 and IL-17A and negative correlations with IL-21 and vitamin D3. Vitamin D3 also has significant negative correlations IL-23 and IL-17A.

## Discussion

It has been proposed that Th17 plays crucial roles in the pathogenesis of proinflammatory disorders including autoimmune diseases via the production of IL-17A [17, 18]. IL-21 and IL-23 are important cytokines which play pivotal roles in development and maintenance of Th17, respectively [19]. Thus, it has been hypothesized that the cytokines may participate in the pathogenesis of OA, as a proinflammatory disease. The results presented here showed that serum levels of IL-17A and IL-23 significantly increased, while IL-21 significantly decreased in OA patients in comparison to healthy controls. Based on the fact that OA is an inflammatory disease, the results demonstrate that IL-17A significantly participates in the pathogenesis of OA. Interestingly, IL-23 the main cytokine required for the maintenance of Th17 lymphocytes, also increased in the patients, hence, it seems that survival and function of these lymphocytes are increased in OA patients. Based on the fact that IL-21 plays key roles in the development of Th17 lymphocytes, and according to the decreased serum levels of the cytokine in OA patients, it could be possible that the increased functions and survival of Th17 (by IL-17A and IL-23) lead to a negative feedback to decrease IL-21 and development of new clones of Th17 lymphocytes.

Previous investigations demonstrated the reactivity of T cells against chondrocyte antigens in OA [20] and also showed that IL-17A induces production of IL-1β, TNF-α and IL-6, the main pro-inflammatory cytokines, in OA. Moreover, the role of these proinflammatory cytokines in production of NO and MMP (matrix metalloproteinase) and reduction of proteoglycan levels has been shown in OA pathogenesis [21–24]. Furthermore, it has been suggested that IL-17A can inhibit proteoglycan synthesis in chondrocytes through NO induction [24]. Based on the aforementioned investigations and the results of the current study, it seems that IL-17A and its maintenance factor (IL-23) may be considered as key factors for induction of inflammation and osteoarthritis in the OA patients. The roles of IL-21, as the inducer of Th17 differentiation, need to be evaluated by additional studies in the context of OA. To the best of our knowledge, serum levels of IL-21 and IL-23 have not evaluated in OA patients, hence, this is the first report regarding the status of IL-21 and IL-23 in OA patients.

Vitamin D3 plays is an immunomodulator which has a significant effects on immune cells via interactions with its intracytoplasmic receptor. Results of the current study demonstrated that serum levels of vitamin D3 were significantly decreased in OA patients in comparison to healthy controls. Based on the immunomodulatory effects of vitamin D3, it seems that declined vitamin D3 is an important factor that may induce inflammation in OA patients. Statistical analysis also confirmed this hypothesis and revealed that serum levels of vitamin D3 have a negative correlation with serum levels of IL-23 and IL-17A in OA patients. Thus, it may be proposed that using vitamin D3 as therapeutic agent in OA patients may reduce immune responses and inflammation. However, previous investigations reported that administration of vitamin D3 is not effective for knee OA [14], but based on our results it may be effective against the Iranian genetic background.

The results also demonstrated that IL-17A and IL-23 have a significant positive correlation while vitamin D3 and IL-21 have a significant negative correlation with WOMAC pain scores in OA patients. It has been documented that IL-17A induces expression of cyclooxygenase 2 (COX2) and prostaglandin E2 (PGE2) production via the activation of MAPKs pathway [25]. It may be hypothesized that IL-17A induces pain in OA patients through the same pathway but this needs to be examined further. Additionally, based on the fact that serum levels of IL-21 and vitamin D3 decreased in OA patients it is possible that this also influences pain. Thus, it seems that more investigations regarding the roles played by these cytokines in the induction of pain in OA patients would shed light on the pathogenesis and clinical symptoms of this disease.

## Conclusions

According to our data, it seems that the IL-17A may play a major role in the pathogenesis of OA and decreased serum levels of vitamin D3 in these patients may be a reason for the increased functions of Th17. Additionally, based on the positive correlation between serum levels of IL-17A and WOMAC pain score, this may open a door for new approaches in the treatment of pain and OA complications. Furthermore, the data suggests that the serum levels of IL-17A can reflect OA development and potentially be considered as a new biomarker for the disease.
